# Presentation of an umbilical cord cyst with a surprising jet: a case report of a patent urachus

**DOI:** 10.12688/f1000research.2-38.v1

**Published:** 2013-02-11

**Authors:** John Svigos, Sanjeev Khurana, Christopher Munt, Sanjay Sinhal, Julie Bernardo

**Affiliations:** 1 Women’s and Children’s Hospital, North Adelaide, SA 5006, Australia; 2 Ashford Hospital, Keswick, SA 5035, Australia; 3 Flinders Medical Centre, Adelaide, SA 5042, Australia

## Abstract

We report a baby with an unusual true umbilical cord cyst detected at 12 weeks gestation which as the pregnancy progressed became increasingly difficult to distinguish from a pseudocyst of the umbilical cord. Concern of the possibility of cord compression/cord accident led to an elective caesarean section being performed at 35+ week’s gestation with delivery of a healthy female infant weighing 2170g. At birth the cyst ruptured and the resultant thickened elongated cord was clamped accordingly. After the cord clamp fell off at 5 days post delivery an elongated umbilical stump was left behind from which a stream of urine surprisingly jetted out from the umbilicus each time the baby cried. A patent urachus was confirmed on ultrasound and the umbilical jet of urine resolved at 4 weeks post delivery after treatment of an Escherichia coli urinary tract infection. At 11 weeks post delivery a laparoscopic excision of the urachus was successfully performed. The baby, now 18 months of age, continues to thrive without incident.

## Introduction

A persistent urachus is a result of failure of involution at 10–12 weeks gestation of the allantois which communicates from the dome of the bladder to the umbilicus. Embryologically, the allantois is an endodermal diverticulum which becomes the urogenital sinus with the cranial portion developing as the bladder. Persistence of the urachus may be partial resulting in an urachal cyst, diverticulum or sinus, or it may be completely patent allowing communication with the bladder ^1^. Just over 100 cases in the neonatal period have been documented after the first report in the 16 ^th^ Century ^2^.

## Case report

**Figure 1.**
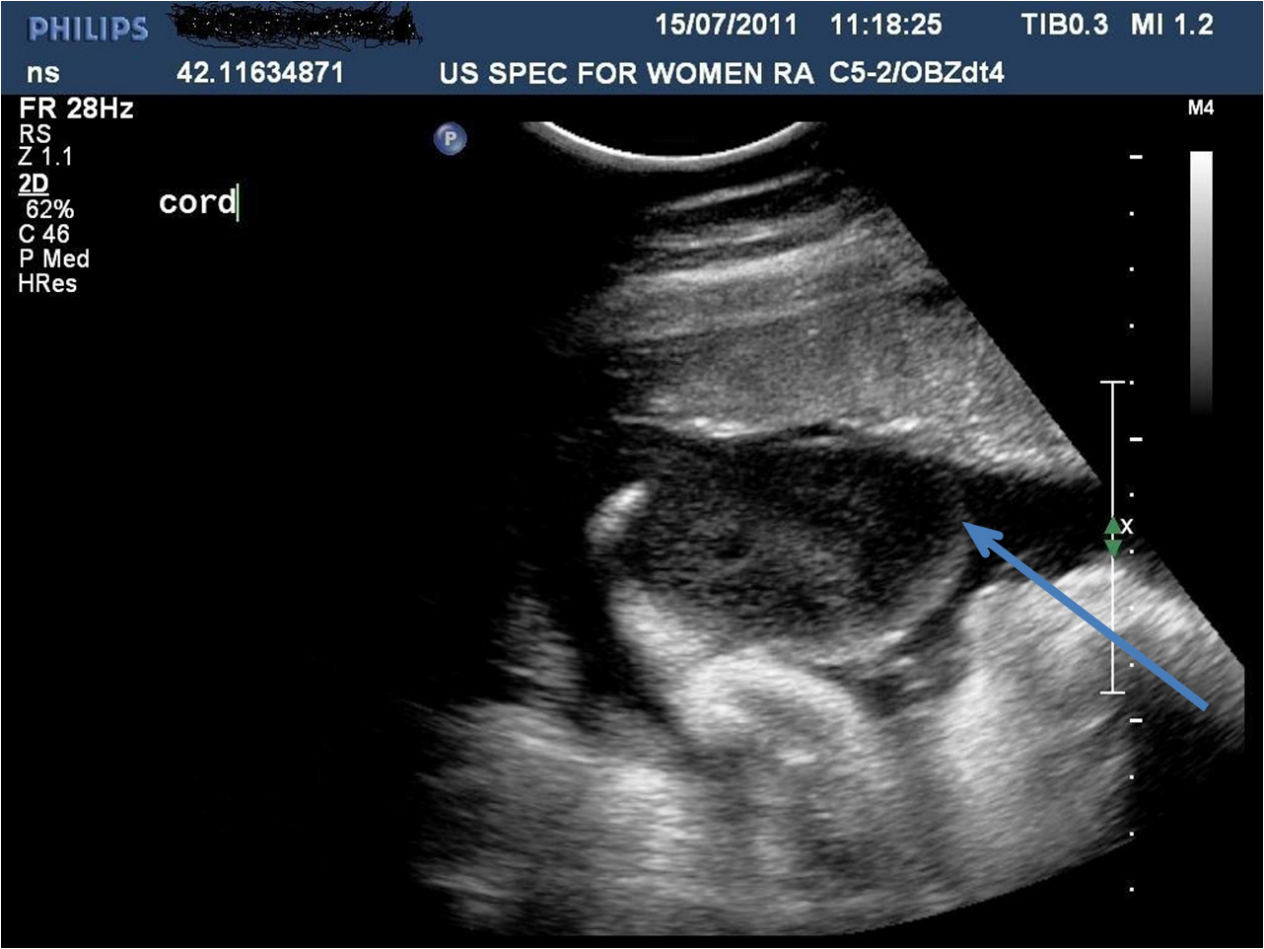
Umbilical cord cyst at 35 weeks gestation with: homogeneous echogenicity? Oedema? Wharton's jelly?

A 36 year old Caucasian woman with a 4 year history of infertility presented at 9 weeks gestation for antenatal care following a successful single embryo transfer after intra-cytoplasmic sperm injection (ICSI). First Trimester screening at 12 weeks gestation demonstrated a trisomy 21 risk of 1:2760 and a trisomy 18 risk of 1:4060. However, detailed ultrasonic examination of the fetus revealed a hypoechoic area on the anterior abdominal wall which was thought possibly to be fluid distending the urethra or an extra-abdominal mass such as an omphalocoele. At 16 weeks gestation a repeat ultrasound examination identified an umbilical cyst, of dimensions 1.7 cm x 1.6 cm x 1.8 cm with vessels coursing around it with no gut present within and a normal anterior abdominal and bladder wall. The uncertainty of the type of cyst and possible reduction in the fetal dimensions required trisomy 18 to be excluded and an amniocentesis revealed a normal XX karyotype. The umbilical cyst increased progressively with advancing gestation, increasing in dimension, to 4.9 x 4.5 x 4.7 cm at 35 weeks gestation (See Figure 1).

On the ultrasound, the cord developed homogeneous echogenicity, which was thought to be due to increased Wharton’s jelly, seen more commonly with pseudocysts of the cord; or due to oedema which was a more sinister sign of possible cord compression/cord accident. In view of the latter, after a course of preoperative maternal cortico-steroids to enhance fetal lung maturity, the patient underwent an elective caesarean section under a combined epidural/spinal anaesthetic block, with delivery of a healthy female infant. Unfortunately, during the process of the delivery the cord cyst ruptured making accurate diagnosis impossible. However the cord was thickened with Wharton’s jelly and was elongated with an umbilical stump with overlying skin up to 2 cm in length. The cord was divided in the standard fashion and a normal placenta was removed.

**Figure 2.**
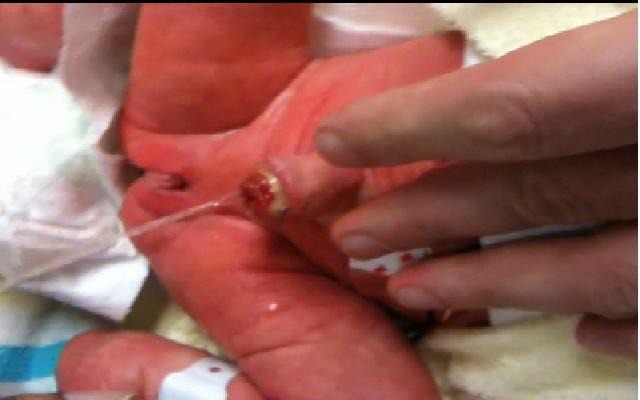
Urine jetting out of the umbilicus giving the impression of a pseudophallus.

On the 5 ^th^ day post-delivery, the cord clamp fell off and each time the baby cried a stream of urine jetted out from the umbilicus (see Figure 2). This phenomenon finally ceased 4 weeks after delivery, following successful treatment of a urinary tract infection. A patent urachus was confirmed on the ultrasound. The very prominent umbilical stump created the appearance of a ‘pseudophallus’ due to bulging from increased intra-abdominal and bladder pressure after each normal micturition.

A successful laparoscopic excision of the urachus was performed 11 weeks after delivery. At surgery, the entire dome of the bladder was seen to be in continuation with the umbilicus rather than the more commonly seen urachal tract. The ureteral orifices in the bladder were not affected by the closure, whilst a micturating cysto-urethrogram performed post-operatively failed to show any evidence of uretero-vesical reflux. The baby was discharged home 36 hours after surgery. The baby had no further urinary tract infections post-operatively and follow-up blood pressure, growth and development were normal at 18 months of age.

## Discussion

The prevalence of umbilical cord cysts detected by ultrasound in the first trimester is of the order of 0.4–3.4%, with a patent urachus being a rare anomaly with an incidence of 1–2.5 per 100,000 ^3^. Whilst umbilical cord cysts are classified as true cysts or as pseudocysts differentiation from each other is often difficult antenatally and a detailed morphology scan and karyotype is recommended as both types of umbilical cysts may be associated with omphalocoele which has an increased incidence of aneuploidy and other fetal anomalies (25–85%) ^4^. True cysts are less common, ranging in size between 4 and 60 mm, are located towards the anterior abdominal wall of the fetus, split the umbilical vessels, contain fluid and if present in the second and third trimesters, are associated with an omphalocoele or patent urachus ^4, 5^.

Pseudo cysts are more common, are usually smaller, and may be located anywhere along the length of the umbilical cord. The umbilical vessels are pushed to one side as a result of local oedema and liquefaction of Wharton’s jelly and may be associated with omphalocoele, hydrops and aneuploidy (trisomy 18) ^4, 5^. Post-delivery the differential diagnosis of an umbilical cord cyst is between an umbilical pseudo-cyst and an allantoic cyst with a patent urachus which can be distinguished by ultrasound. Ultrasound shows the channel communicating from the umbilicus to the bladder. Bladder outlet obstruction can be excluded by a micturating cysto-urethrogram preoperatively or by an intra-operative cystoscopy. The contemporary treatment of a patent urachus is by laparoscopic surgical excision ^6^ whilst other urinary tract abnormalities (including urachal cysts, fistulae and diverticulae) are investigated and treated appropriately ^7^. Post-operatively these babies are given antibiotic prophylaxis until a normal ultrasonogram of the urinary tract is demonstrated.

## Conclusion

Antenatal ultrasound detection of an umbilical cyst, particularly if located close to the anterior abdominal wall of the fetus should stimulate a search for a patent urachus post-natally. Laparoscopic surgical correction is the treatment of choice for a baby with a patent urachus. Reduced post operative morbidity and length of hospital stay are important advantages in comparison to the open surgical approach with both methods being equally successful in closing a patent urachus.

## Consent

Written informed consent for publication of clinical details and clinical images was obtained from the mother of the neonate.
