# Supplementary material for: Presentation of an umbilical cord cyst with a surprising jet: a case report of a patent urachus
Source: F1000Res. 2013 Feb 11;2:38. [Version 1] doi: 10.12688/f1000research.2-38.v1 (PMC3814933; doi:10.12688/f1000research.2-38.v1)
Supplement: Peer review history

# Peer review: Referee response for version 1

**Published:** 2013-05-02

**Authors:** Henry Murray

**Affiliations:** 1 Maternal Fetal Medicine Unit, John Hunter Hospital, New Lambton Heights, NSW, Australia

This is an excellent case report with appropriate referencing and balanced discussion. I would have liked to see in the conclusion a comment about the lack of evidence on antenatal scanning of the patent urachus. For the reader, this would further highlight the need for the postnatal assessment of a baby with the antenatal diagnosis of cord/umbilical cyst(s), even if the antenatal scan suggested normal bladder anatomy.

I have read this submission. I believe that I have an appropriate level of expertise to confirm that it is of an acceptable scientific standard.

# Peer review: Referee response for version 1

**Published:** 2013-02-27

**Authors:** George Condous

**Affiliations:** 1 Acute Gynaecology, Early Pregnancy and Advanced Endosurgery Unit, Nepean Centre for Perinatal Care, Nepean Clinical School, University of Sydney, Sydney, New South Wales, Australia

I think this case presentation is well written and appropriately referenced. 

I have one question for the authors:

In retrospect, do the authors believe that early delivery at 35+ weeks gestation by LSCS would have been justifiable if they had have made the diagnosis antenatally? And further to this, is there an increase in perinatal mortality if these babies with a patent urachus are allowed to delivery at term? 

I think the Conclusion should also be changed to read: 

'Antenatal ultrasound detection of an umbilical cyst, particularly if located close to the anterior abdominal wall of the fetus should alert the clinician to the possibility of an underlying patent urachus. Once diagnosed post delivery, the laparoscopic approach is the preferred surgical route.'

I have read this submission. I believe that I have an appropriate level of expertise to confirm that it is of an acceptable scientific standard.
